# A retrospective cohort study of patients treated with anti-tuberculous therapy for presumed ocular tuberculosis

**DOI:** 10.1186/s12348-017-0141-4

**Published:** 2017-12-04

**Authors:** Erika Marie Damato, Sarah Dawson, Xiaoxuan Liu, Chandoshi Mukherjee, John Horsburgh, Alastair K. Denniston, Edward Moran, Martin Dedicoat, Philip Ian Murray

**Affiliations:** 1 Birmingham and Midland Eye Centre, City Hospital, Sandwell and West Birmingham Hospitals NHS Trust, Birmingham, B18 7QH UK; 2 Sandwell General Hospital, Lyndon, West Bromwich, West Midlands B71 4HJ UK; 3 Department of Ophthalmology, Queen Elizabeth Hospital Birmingham, University Hospitals Birmingham NHS Foundation Trust, Birmingham, UK; 4 Institute of Inflammation and Ageing, College of Medical and Dental Sciences, University of Birmingham, Edgbaston, Birmingham, B15 2TT UK; 5 Heartlands Hospital, University Hospital Birmingham NHS Foundation Trust, Birmingham, UK; 6 Birmingham Chest Clinic, University Hospitals Birmingham NHS Foundation Trust, Birmingham, UK

**Keywords:** Uveitis, Anti-tuberculous therapy, Tuberculosis, Interferon-gamma release assay

## Abstract

**Background:**

Uveitis involving the posterior segment is a significant and potentially blinding condition. The diagnosis and treatment of patients with uveitis associated with tuberculosis remains controversial, and commonly, patients are systemically well. Use of the interferon-gamma release assays has added to the controversy, as the significance of a positive test may be uncertain. We aim to report the outcomes of anti-tuberculous treatment in a cohort of patients treated in Birmingham, for presumed “ocular tuberculosis”, based on clinical findings, systemic assessment and specific testing for tuberculosis.

**Results:**

We found that in our cohort of 41 patients treated between 2010 and 2014, the majority achieved disease-free remission, even in cases where anti-tuberculous treatment was delayed.

**Conclusions:**

Despite controversy, this study strongly supports the use of anti-tuberculous therapy in such patients and highlights the need for formal prospective trials and treatment protocols.

## Background

Intraocular inflammation, generally termed “uveitis”, may result from a hugely diverse range of causes, both infectious and non-infectious. Uveitis is classified by the predominant anatomical site of inflammation within the eye [1], the cause and whether or not it is related to an infectious agent or an underlying systemic condition [2]. Uveitis is an important cause of visual loss, often affecting the working age population [3, 4].

Uveitis related to tuberculosis is a well-recognized clinical entity, with an extensive literature dating back more than 100 years [5]. In some parts of the world, tuberculosis is one of the principle causes of uveitis [6–9].

Currently, a large proportion of patients in the developed world is described as having “non-infectious”, “idiopathic” or “undifferentiated” uveitis [10, 11]. Often no underlying systemic association or infection is identified despite extensive investigation. If such patients have significant or “sight-threatening” uveitis, they are generally treated with systemic corticosteroid, and sometimes systemic immunosuppression, including biologics [12, 13]. Such therapy is aimed at preventing relapses, as each relapse carries a risk of irreversible visual loss, morbidity and ocular complications [14].

The association between uveitis and “latent” or occult tuberculosis is not new yet has become increasingly relevant. Many patients display clinical appearances identical to those seen in patients with active TB, without demonstrating systemic manifestations of infection. Ocular appearances associated with TB are heterogeneous, making diagnosis challenging, and include (among others) choroiditis, serpiginous-like choroiditis, granulomatous uveitis, retinal vasculitis and intermediate uveitis [15]. In addition, the condition known as “Eales” disease, a bilateral occlusive retinal vasculitis, with minimal inflammation and often complicated by vitreous haemorrhage, is observed more frequently in TB-endemic populations.

A large proportion of the global population will have immunological evidence of latent TB, and consequently, a significant proportion of patients with ocular inflammation will have evidence of latent TB, whether or not it is causal.

Studies investigating intraocular samples provide support for a central role of TB. Analysis of the aqueous humor, vitreous gel or epiretinal membranes using polymerase chain reaction (PCR) methods [16–20] demonstrates that a higher proportion of samples from uveitis patients are positive for the TB genome compared with samples taken from patients in a similar population undergoing surgery for other reasons, and a positive PCR result correlates with a clinical response to anti-tuberculous treatment (ATT) [19, 20].

Traditionally, the tuberculin skin test (Mantoux) has been used to determine whether a patient is infected with TB. The test may be affected by multiple factors, making it hard to determine its significance. More recently, interferon gamma release assays (IGRAs) have added significant controversy to the diagnosis and management of “ocular TB” [21–26]. Such assays, including the T-spot or QuantiFERON GOLD tests rely on the observation that T-cells will release interferon gamma when exposed to a specific TB antigen. The test is highly specific and not affected by previous BCG vaccinations. A positive IGRA test usually indicates that a patient has been exposed to TB; however, it cannot distinguish between latent infection and active disease.

Multiple recent reports support the use of anti-tuberculous therapy (ATT) in patients with a consistent uveitic phenotype and positive TB investigations [27–30]. There is a lack of consensus regarding treatment indications, treatment regimens, investigation protocols and what constitutes a successful outcome. Even the diagnosis of “ocular TB” is not standardized [31].

Importantly, the way in which patients are managed is believed to differ significantly between units, with some uveitis specialists unable to persuade the respiratory physician of the relevance of tuberculosis, whilst other specialists are able to access anti-TB treatment easily and as a result have a very low treatment threshold [32]. Some uveitis specialists elect to refer patients for ATT only if the uveitis is severe, whilst continuing to treat “mild” recurrent anterior uveitis with topical steroid.

The principle indication for using ATT in uveitis is to treat the underlying systemic drive, which is believed to come from occult infection, antigenic mimicry or a hypersensitivity-type reaction to TB antigen. A secondary indication may be to ensure that systemic immunosuppression is safe in the face of a positive T-spot result. This is especially relevant for patients started with anti-TNF therapy [33].

The Birmingham and Midland Eye Centre is located in an inner-city part of Birmingham where the large surrounding population comprises of ethnically diverse communities with a majority of people originating from Southeast Asia, especially countries such as Pakistan, India and Bangladesh, where the prevalence of TB is high.

### Aims and objectives

The aims of this study were first, to define and report the treatment outcomes of patients, who received anti-tuberculous therapy for uveitis, and second, to characterize the patient cohort seen at the Birmingham and Midland Eye Centre who were diagnosed as having ocular TB.

## Methods

This was a retrospective evaluation of patient notes. Ethical approval was not required as the project was deemed to be a service evaluation.

Using the regional TB disease register obtained from the regional infectious diseases department, patients who had been diagnosed as having uveitis related to TB and who were started on ATT between 2010 and 2014 were identified. Patients who completed a course of treatment and were followed up for at least 12 months were included in the study.

A retrospective analysis of patient notes was undertaken. Ethical approval was not required as this was deemed to be a service evaluation and made use of anonymized retrospective patient information. Data pertaining to patient characteristics, uveitis phenotype, vision, duration of uveitis prior to therapy, date of commencement of ATT, treatment details, duration of follow-up and disease activity were collected. Data were also collected regarding systemic investigations for TB.

At presentation to the uveitis service, patients underwent a full ophthalmic examination and were assessed with regard to the site and severity of their uveitis, using the SUN criteria [1]. A full systemic workup was undertaken, and patients underwent an extensive panel of investigations, with the aim of excluding other causes. All underwent chest radiograph or CT scan, with most also undergoing Mantoux testing or interferon gamma release assay, usually using T-spot. Investigations were not protocolized, and patients were investigated appropriately in an individualized fashion.

A consistent ocular phenotype, together with findings on chest imaging and/or positive T-spot or Mantoux testing, with the exclusion of other causes of uveitis, led to a diagnosis of presumed ocular TB.

Following this diagnosis, patients were referred urgently to the respiratory team where they were investigated further as necessary. Throughout the course of the study, the availability and use of interferon gamma release assays increased. This meant that some patients were not diagnosed with TB-related uveitis until a significant period of ocular inflammation had lapsed.

In most patients, disease activity was observed as a number of relapses, rather than continual inflammation. Treatment with anti-inflammatory medication, mainly corticosteroids, was used. These were administered topically, peri-ocularly or systemically. Relapse on withdrawal of therapy warranted repeat treatment.

Ocular disease activity was quantified using the number of “flare-ups” of uveitis. A uveitis “flare” was defined as the need for augmentation of treatment, with high-dose oral steroid (usually at least 40 mg daily tapered over several weeks), intravenous steroid, periocular steroid or hourly topical steroid. Where possible, “flare rate” prior to ATT was defined as the number of flare-ups per unit time of disease. Calculating a “flare rate” was not possible if a patient was treated with ATT immediately or had a very short duration of disease prior to ATT.

### Statistical methods

Data were reviewed using descriptive statistics. The “time to flare” post-treatment was calculated using Kaplan-Meier survival curves. Remission was defined as the absence of flare-ups for at least 6 months whilst long-term remission was defined as at least 12 months of disease quiescence after completion of therapy. Flare rates were compared before and after therapy using the Wilcoxon signed rank test.

## Results

### Demographics

A total of 54 patients were identified as having uveitis related to ocular TB and for whom treatment was recommended. Of these, 41(76%) patients completed therapy and complied with follow-up for at least 12 months. The remainder either did not complete therapy, did not comply with follow-up or both. The characteristics of the initial patient cohort are described in Table 1. The majority of patients were male, of Asian or Black ethnicity with a mean age of 44 years.

**Table 1:** Characteristics of the patient cohort

Gender (number of patients (%))		
Male	33	61%
Female	21	39%
Age, years, mean (range)	44 (17–69)	
Ethnicity (number of patients (%))		
Asian	31	57%
Black	11	20.37%
Not specified	12	22.22%
Diabetic (number of patients (%))	8	14.81%
Site of uveitis (number of patients (%))		
Anterior	6 (11.11%)	
Intermediate	15 (27.78%)	
Posterior	12 (22.22%)	
Panuveitis	19 (35.19%)	
Other	2 (3.70%)	
Bilateral disease (number of patients (%))	41.58(77%)	
Uveitis features present (NB some patients may have had more than one feature)		
Nodular scleritis	1	
Choroiditis	7	
Granulomatous anterior uveitis/ mutton fat KP	7	
Retinal vasculitis	5	
Panuveitis	13	
Intermediate uveitis	18	
Not recorded	3	
Maintenance treatment prior to ATT	Number of patients (%)	
Unknown	8 (14.80)	
Nil	13 (24.10)	
Topical steroid alone	20 (37)	
Oral steroid alone	3 (5.60)	
Oral steroid and immunosuppression	2 (3.70)	
Topical steroid and immunosuppression	1 (1.90)	
Topical and systemic steroid	7 (13)	

### Ocular disease

Uveitis was predominantly bilateral with most having disease affecting the posterior segment. Isolated retinal vasculitis, including the “Eales’ phenotype”, was categorized as posterior uveitis. A wide range of uveitic phenotypes was observed, including granulomatous anterior uveitis, retinal vasculitis, nodular scleritis, choroiditis and intermediate uveitis.

The date of first presentation with uveitis preceded the diagnosis of TB-related eye disease in the majority, with four patients starting ATT at the same time as their uveitis was diagnosed. The duration of disease prior to ATT ranged from 8 to 4495 days (more than 12 years) with a median of 12 months. Prior to ATT, 31 patients were on maintenance therapy for uveitis as described in Table 1.

### Investigations to support the diagnosis of active or latent tuberculosis

Data regarding the results of investigations arranged from the uveitis clinic are shown in Table 2. It is seen that a large proportion of patients (29) were referred for ATT for ocular TB mainly on the basis of a positive Mantoux and/or T-spot result, with normal chest X-ray imaging and no systemic symptoms.

**Table 2:** Investigations to support TB

	Number of patients
No details available	4
Chest CT changes	4
With positive T-spot or Mantoux test	3
Positive Mantoux alone	15
Positive T-spot alone	12
Positive T-spot and Mantoux test	2
Typical X-ray changes present	6
With a positive T-spot and/or Mantoux test	6
Systemic symptoms present^a^	11
With a positive T-spot and/or Mantoux test	7
With consistent CT or Chest X-ray findings	3

^a^One patient underwent a bone marrow biopsy which confirmed mycobacterium tuberculosis

### ATT regimens, duration and compliance

Of the 54 patients in whom treatment was recommended, 7 (13%) patients did not comply. The remaining 47 patients received a course of ATT. Duration of therapy ranged from 4 to 12 months with a mean of 6.8 months. Treatment regimens varied; however, all patients received rifampicin, with 9 receiving rifampicin, isoniazid and pyrazinamide (RHZ) and 27 received RHZ and ethambutol (E). See Table 3.

**Table 3:** Treatment regimens

R alone	2
R + E	2
R + Z	1
RHZ	9
REZ	3
RHZE	27
Anti-TB (unknown specific treatment)	3
Additional prednisone	37
Non-compliant	7
Mean duration of therapy	6.8 months (range 4–12)

*Rifampicin R, isoniazid H, pyrazinamide Z, ethambutol E

### Disease activity following completion of therapy

Of the 47 patients who complied with ATT, follow-up data were available for 41, with the remaining patients not attending follow-up or having missing data. Duration of clinic follow-up ranged from 20 to 2192 (median 557) days with five patients being lost to follow-up or who had moved elsewhere. Patients completing therapy were instructed to attend eye casualty in the event of flare-up, and it was assumed that their disease remained quiescent if no attendances were recorded. The mean clinic follow-up was 808 days.

**Fig. 1:**
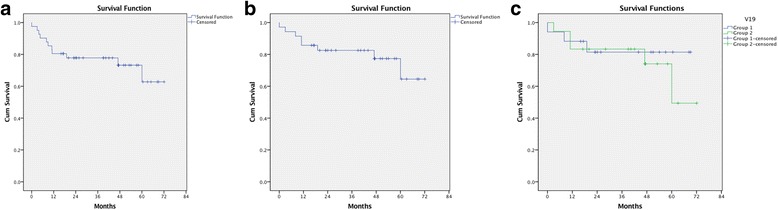
Kaplan-Meier Survival curves showing: (**a**) time to flare-up for patients with post ATT followup data, (**b**) time to flare-up for patients with both pre and post ATT data and (**c**) time to flare-up for patients in group 1 and group 2 where group 1 were treated with ATT within 8 months of their first uveitis attack and group 2 were treated more than 8 months after their first uveitis attack

A Kaplan-Meier survival curve for the 41 patients with post-ATT follow-up data is shown in Fig. 1a. The survival curve shows 90% of patients (39) were flare free 6 months following ATT treatment and 80% (33) remained quiescent for 12 months.

A survival curve including patients for which both pre- and post-treatment data was available is shown in Fig. 1b. This highlights that 94% of patients were flare free at 6 months and 86% were flare free at 12 months.

The data set was then divided into two groups, those patients who were treated with ATT within 8 months of their first uveitis episode (group 1) and the remaining (group 2) who had had a diagnosis of uveitis for more than 8 months. This was to see whether patients with a longer duration of uveitis were less likely to respond favorably to ATT. Kaplan-Meier and log-rank tests (shown in Fig. 1c) were used for analysis. No significant difference in the time to flare was found (*P* = 0.565 log-rank) between the two groups.

The monthly flare rate pre-ATT treatment was calculated from the first uveitis episode to initiation of ATT. It was assumed the nine patients with a very short time to treatment (< 4 months) would have had just one flare in the time period. Post-treatment flare rates were calculated from time of ATT completion to last day of data collection (December 2016). Flare rates after ATT were significantly lower than those before (Wilcoxon signed rank *P* = 0.000).

The data was further split into two groups; group 1 included patients treated within 8 months of their first uveitis flare. Group 2 included those who were treated more than 8 months after their first uveitis flare. Flare rates post-ATT treatment for both groups were significantly lower than pre-ATT (Wilcoxon signed rank *P* = 0.000, *P* = 0.000 respectively).

To assess whether the short time to treatment following first uveitis episode had an overall effect on the significant difference in pre- and post-ATT flare rates, the nine patients with less than 4 months follow-up were excluded and the data re-analysed. The post-ATT flare rates remaining significantly lower following the exclusion of these patients (*P* = 0.000 Wilcoxon signed rank). Of the nine patients excluded, all were flare free at 12 months.

Disease flare rates were also calculated using rates per person year (PPY). Prior to ATT treatment, the flare rate was 0.69 while after ATT, this reduced to 0.14. The difference was statistically significant (*P* = 0.000 mid-*P* exact).

## Discussion

The principal finding from this study is that ATT in a real-life clinical setting appears to significantly reduce the number of flare-ups and to enable long-term remission in patients with presumed ocular tuberculosis. This beneficial effect appears to persist even in patients who experienced a long delay between the onset of uveitis and the diagnosis of ocular TB.

The patient cohort is diverse and strict diagnostic criteria are absent, requiring a high degree of clinical judgment and collaboration between specialists when treating such patients.

The study has limitations in view of the “real-life” clinical setting. Many were lost to follow-up or non-compliant with ATT. “Flare rate” was used as a measure of disease activity but may not accurately reflect disease in patients with ongoing activity, where uveitis is chronically uncontrolled, or being suppressed with corticosteroid. ATT was not standardized, and consequently, patients received a range of regimens. The majority however received at least 6 months of therapy. There is evidence to support treating patients for at least 9 months [29] although a wide range of treatment regimens and durations is reported in the literature.

Recent studies investigate therapeutic outcomes and the beneficial effects of ATT. One meta-analysis concluded that ATT in the management of patients with presumed ocular TB enabled remission in 84% [30]. Another large analysis explored whether any factors were associated with a poorer outcome and found that prior immunosuppression and/or a high absolute QuantiFERON GOLD level were associated with poorer resolution of inflammation post-ATT or an ongoing need for systemic steroid [34].

This study highlights challenges and controversies in defining outcomes in such cohorts. We attempted to look at therapeutic outcomes in a number of ways, including comparing flare rates pre and post, reporting time to flare using Kaplan-Meier survival curves and also calculating “flare rates” before and after treatment. We also recognize that such measures may introduce bias and favour a benefit in patients with a short period of disease prior to the use of ATT. For this reason, we compared results between patients with a long duration of disease and a shorter duration of disease prior to ATT. Despite some patients experiencing significant delays in being started on ATT, we showed that such patient still displayed a clear benefit from treatment.

We did not report visual outcomes or complications. Whilst we show that there is a significant treatment benefit, even when duration of uveitis is long, patients with longer disease courses prior to ATT will be more likely to suffer visual loss and complications. Therefore, early treatment should be recommended [35].

## Conclusions

In conclusion, this retrospective study shows that despite the unresolved controversies regarding diagnosis, the relevance of interferon gamma assays and what exactly ocular tuberculosis is, if patients have a consistent uveitis phenotype and other uveitic causes are excluded, then ATT is likely to have a beneficial effect.
